# Training of Lived Experience Workforces: A Rapid Review of Content and Outcomes

**DOI:** 10.1007/s10488-022-01232-z

**Published:** 2022-11-10

**Authors:** Jessica E. Opie, Siân A. McLean, An T. Vuong, Heather Pickard, Jennifer E. McIntosh

**Affiliations:** 1 School of Psychology & Public Health, La Trobe University, Melbourne, 3056 Australia; 2 Self Help Addiction Resource Centre, Melbourne, 3163 Australia; 3 The Bouverie Centre, School of Psychology & Public Health, La Trobe University, Melbourne, 3056 Australia

**Keywords:** Lived experience workforce, Peer workforce training, Carer workforce training, Mental health, Alcohol and drug

## Abstract

Recently, the lived and living experience (LLE) workforce in mental health and alcohol and other drugs (AOD) sectors has expanded. Despite widespread benefit of this inclusion, some LLE practitioners have encountered personal and professional challenges in their workforce roles. An essential avenue to address these challenges is through provision of training to ensure adequate LLE role preparation, and to support integration of LLE workforces within mental health and AOD settings. We aim to understand the primary components applied in LLE training programs (i.e., content and methods), the outcomes from program participation, and to summarize observed patterns between training components and outcomes. This rapid review utilized a systematic methodology following the Preferred Reporting Items for Systematic Reviews and Meta-Analyses guidelines to synthesize existing literature on training programs for service users or carers/family in lived experience roles, in the mental health and AOD workforce. We searched CINAHL, PsycINFO, Medline, and Web of Science databases. We identified 36 relevant studies. Findings indicate short- and long-term impacts of training participation for this emerging workforce, with the most promising outcomes being increased professional knowledge and skills and improved personal psychosocial wellbeing and trauma recovery. Other positive training outcomes included high trainee satisfaction, increased application of training skills, and employment/education opportunities following training completion. Gaps and training limitations were noted in relation to the training content/delivery, trainee reservations, and personal barriers to training participation or completion. In response to program benefits and limitations investigated, we present recommendations for improving training processes for this workforce.

**Supplementary Information:**

The online version contains supplementary material available at 10.1007/s10488-022-01232-z.

Lived experience refers to people with direct experience-based knowledge and understanding of mental health or alcohol and other drug (AOD) difficulties, service system use, and recovery, it also relates to the experience of those who support another throughout their journey (Byrne et al., 2021a). The importance of both voluntary and paid contributions made by people with service user or carer lived experience of mental health and AOD services is increasingly recognized. Workforce roles include peer support workers, consultants, educators, and advocates. In the context of this paper, we collectively refer to these roles as the lived and living experience (LLE) workforces, appreciating that those in the AOD sector include ‘living experience’ workers (Our Future Project Partnership, 2021). Growth in these workforces has been driven by a dominant shift to recovery-oriented perspectives of care (Le Boutillier et al., 2011; Minshall et al., 2021) and reflects the unique role the LLE workforces can play in supporting recovery of others through the sharing of experience, including of treatment and recovery, instilling hope, role modelling, and minimizing distance between service users and practitioners (Davidson et al., 2006; Otte et al., 2020). Positive individual effects for those taking on these workforce roles have been observed. These relate to personal recovery including engagement in self-care behaviors and reduced need for service engagement, increased self-confidence and self-esteem, and enhanced social functioning (du Plessis et al., 2020; Meagher & Naughtin, 2018; Walker & Bryant, 2013).

The effectiveness of LLE workforce delivered interventions and support for improving symptoms and recovery-related outcomes for people with mental illness and AOD problems has been examined through research trials and qualitative exploration. Meta-analytic evidence suggests that LLE-delivered interventions for mental health problems are efficacious for the service user at the psychosocial self-management level, for fostering patients’ hope, recovery, and empowerment, and for improved quality of life (Cabassa et al., 2017; Fuhr et al., 2014; Lloyd-Evans et al., 2014). Similarly, in AOD settings, there is some evidence for the benefits of LLE workforce inclusion in service delivery, although the literature is less well developed than for mental health settings. A systematic review identified advantages of employing service users with lived experience in AOD contexts, including widespread perceptions of the LLE member having knowledge and skills to perform effectively, to be more understanding due to their shared lived experience, and able to use their journey to aid recovery for other service users (Goodhew et al., 2019). Another systematic review examined studies in which service user LLE workers were engaged in service delivery for individuals with mental illness (with and without substance use problems) and found moderate support for reduced inpatient service use, improved relationships with providers and better engagement with care, as well as higher levels of empowerment and hopefulness for recovery (Chinman et al., 2014). Although research on family/carer workforces in mental health and AOD is scarce, qualitative studies have found that mental health services generally benefited from having LLE workers with carer experience within their teams (Ehrlich et al., 2020). Further, recipients of family/carer support have reported positive benefits from engagement with these workers through provision of emotional and practical support, sharing of similar experiences, and demonstration of empathy with other carers/family members seeking services (Visa & Harvey, 2019).

Despite these identified benefits of LLE workforce inclusion in mental health and AOD services, some LLE practitioners have encountered personal and professional challenges in their workforce roles within mental health settings. At the organizational level, commonly reported concerns include lack of familiarity with navigating a new workplace and organization, role definition ambiguity, insufficient training, and marginalization and isolation from other professionals (Byrne et al., 2019; Kemp & Henderson, 2012; Moll et al., 2009; Moran et al., 2013; Scanlan et al., 2020; Walker & Bryant, 2013). Personal complexities include challenges in making the transition from service user to service provider or supporter, and balancing professional roles while managing personal health (Moll et al., 2009; Moran et al., 2013). Similar challenges for LLE workers have been recognized in drug and substance abuse treatment settings. For example, a systematic review found that workers have encountered difficulties integrating in their work environment, unclear job descriptions, inadequate role preparation through insufficient training and perceived lack of support in the role, and challenges juggling the dual role of service provider and service user (du Plessis et al., 2020).

## Training Needs

One essential avenue to address some of these challenges is through provision of training to ensure adequate preparation for LLE workforce roles, and for integration of LLE workforces within mental health and AOD settings (e.g., Burr et al., 2020; Byrne et al., 2021b; Moran et al., 2013). Research has shown that having adequate training was associated with greater job satisfaction for LLE workers (Cronise et al., 2016). Furthermore, momentum is building within service systems for appropriate training programs. For example, the recently released report of the Royal Commission into Victoria’s Mental Health System in Australia recommended that “all lived experience workers should have access to a minimum, standardized level of lived experience training” (State of Victoria, 2019, p. 442). Specificity is now needed as to the most appropriate content for inclusion in training programs together with appraisal of any existing evidence for the impact of training on professional and personal outcomes for trainees. With these questions in mind, this study aimed to review published empirical literature examining training programs or packages for LLE workforce roles. The research questions were 1) What are the common content topics and methodologies included in training for LLE workforce roles; 2) What are the professional and personal outcomes for trainees from participating in LLE workforce training; and 3) How do outcomes align with training content and methodologies?

## Method

This rapid review was informed by the Preferred Reporting Items for Systematic Review and Meta-Analysis (PRISMA; Moher et al., 2009) guidelines and followed the Cochrane Rapid Reviews guidelines, and was conducted using systematic search strategies. Given that this is a rapid review, it was not registered. See Online Appendix 1 for completed PRISMA checklist (Page et al., 2021).

### Eligibility Criteria

Eligibility criteria were a) peer-reviewed publication in English; b) cross-sectional, prospective, experimental, quasi-experimental or qualitative studies that examined training for a workforce role (paid or volunteer) for trainees with lived experience (personal or in a carer/family role) of a mental illness or AOD problem, or lived experience of treatment settings as a service user or carer/family. Since this is the first known review of this area, studies with varying designs were included to ensure all relevant literature pertaining to workforce training in mental health and AOD settings was identified. Studies were excluded if they did not contain original data, such as reviews or commentaries. The publication data range was restricted to 2000 onwards, given the recency of the LLE literature.

### Search Strategy

A systematic search of the CINAHL, PsycINFO, Medline and Web of Science electronic databases was conducted in July 2021 to address the concepts of (a) lived experience (peer, consumer, service user, carer/family), (b) workforce (worker, support), (c) training (education, learning), and (d) mental health or AOD contexts. Keyword and subject heading searches were conducted. See Online Appendix 2 for a detailed search strategy for each database. All search terms were co-developed alongside a senior health science librarian.

### Study Selection and Data Extraction

Following deletion of duplicates, each paper was independently screened for eligibility by two researchers across title/ abstract and full-text. Discrepancies were resolved by discussion. Covidence software (Covidence systematic review software) was used to facilitate the screening process. Data extraction was conducted independently without duplication. Data were recorded on a standardized form in accordance with the data shown in Tables 1, 2 and 3.

**Table 1:** Sample, study characteristics, training content, and findings from included quantitative studies

Study (Year) Country	N (% female) Mean age (SD)	Population; position level (service user/carer)	Training purpose/type (Role)/duration (Total hrs)	Training content training delivery methods	Relevant post training findings
Cleary (2009) Australia	NR (66% female) *M*_*age*_ = 43 (10.7)	Dual diagnosis—substance use in people with mental illness) (service user)	PSW training Current (paid) 5 h training	Content: Prevalence of substance misuse, reasons for substance misuse, substance misuse, current therapeutic treatment approaches, referral services, management strategies Methods: Structured presentations, interactive group exercises	Enhanced: 1. Understanding of link between adverse life circumstances & drug use; 2. Sympathy toward people using drugs; 3. Drug use knowledge. All thought program well organised; majority mostly/very satisfied & would recommend; majority found program interesting/very interesting & useful/very useful
Compton (2013) USA	14 (NR) NR	Mental health; entry level (service user)	PSW training NR (NR) 5-day training	Content: 1. Training orientation (e.g., employee code of conduct, de-escalation, & emergency procedures); 2. Core topics (e.g., recovery, relationships that promote recovery, effective listening); 3. In-service trainings	Significant improvements in knowledge & self-efficacy for working in community navigation role
Crisanti (2016) USA	37 (49% female) NR	Mental health & AOD; Entry and mid-level (service user/carer training NR)	PSW training NR (NR) 1 day training	Content: 1. Evidence-based treatment of trauma and/or substance abuse; 2. Increase empathy and understanding of trauma and substance abuse; 3. Describe program, Seeking Safety (SS); 4. Provide assessment and treatment resources; 5. Applying SS for specific populations Methods: 1. Discuss implementation issues; (2) Conduct experiential learning exercises	PSWs and BHPs reported high satisfaction and comfort; equivalent benefit of training for both workers; greater improvement in counselling ability for PSWs
Cronise (2016) USA	597 (64.8% female) NR	Mental health (level & service user/carer training NR)	PSW training Current (paid) 20–80 h of training	Content: 1. Developing peer relationship; 2. Providing peer support; 3. Policy, legislation, advocacy, & rights; 4. Recovery concepts, categories, & principles; 5. Traditional mental health & rehabilitation services; 6. Administrative, supervision, & workplace-related information; 7. Alternative healing & wellness; 8. Pre-crisis & crisis support	Most felt training amount sufficient to complete job. Most agreed/strongly agreed they have job responsibilities that reflect their training and lived experience. Most reported having to complete continuing education. 29% reported receiving 20–40 h/yr ongoing training
Cunningham (2020) USA	575 (62.6% female) NR	Mental health (entry level NR)	PSW training NR (NR) 80 h across 4 wks	Content: 1. Foundations of recovery; 2. Practical aspects of employment in peer support; 3. Integrated health care, selected laboratory skills (e.g., measuring blood pressure), clinic operations, & association between behavioural health & morbidity/mortality	Trainees with psychiatric disability dropout: 25 1% greater among men than women. Mental-illness-only dropout: 17.4%, comparable among men and women Dropout greater among men with psychiatric disability than men with mental illness only. Dropout similar for women across groups
Gammonley (2001) USA	30 (56.7% female) *M*_*age*_ = 39.3 (NR)	Mental health; entry level (service user)	PSW training Future (volunteer) 2 h wkly training over 6 mos (30 h)	Content: 1. Peer helping & advocacy principles; 2. Skill development (e.g., listening & interviewing); 3. Article writing, public education, developing special projects, state, & local advocacy groups (i.e., Americans with Disabilities Act) Methods: Structured presentations, role-play, interactive group exercises, homework tasks	Advocacy skills: Significant increase in phone inquiries about community resources over time, significant reduction in frequency of "discussing accommodation needs with a provider" from baseline to post-training; return to baseline level at 6 mo f/u Significant improvement in satisfaction with QoL. Significant improvement in educational or employment involvement
Hegedüs (2021) Switzerland, Germany	103(67.6% female) *M*_*age*_ = 44.3 (8.89)	Mental health; entry level (service user)	PSW training Future (any capacity) 1.5 yrs	Content: 1. Promoting health and well-being; 2. Trialogue; 3. Empowerment in theory and practice; 4. Experience and participation; 5. Perspectives and experiences of recovery; 6. Independent peer advocacy; 7. Self-exploration; 8. Recovery-based assessment and planning for people in crisis; 9. Peer support; 10. Teaching Methods: 40 h plus 150 h practical training	Significant increase in personal recovery, hope, introspection, stigma resistance and self-efficacy from T1-T2. NS change in mental health related QoL. Post training: Increase in employment. Having a main income from any employment did not change significantly between T1 and T2. Participants whose last inpatient stay was 0–1 yr before training showed lower stigma, & self-efficacy at T1 than participants with 2 + yrs since last inpatient stay
Hoagwood (2018) USA	318 (95% female) *M*_*age*_ = 45.8(9.7)	Mental health; entry level (carer)	FPA training Future (any capacity) 40 h in-person group & 12 × 1 h f/u calls (52 h)	Content: 1. Skill development (e.g., forming family working relationships; assessing family needs); 2. Knowledge on childhood mental disorders, diagnostic processes, treatments, & service options	Post-training: Significantly higher knowledge & mental health service self-efficacy
Horwitz (2020) USA	444 (140 web; 304, in-person) (NR) NR	Mental health; entry/mid-level (carer)	FPA training Future & current roles (paid) 14 × 1 h online modules & 5 day in-person course	Content: 1. Family peer support services & FPA role; 2. Family-led care; 3. Power of lived experience; 4. Embracing family culture; 5. FPA effective communication; 6. FPA engagement strategies; 7. Learning about families: Strengths, needs & culture; 8. Creating family support plan; 9. FPA empowerment strategies; 10. Developing effective partnerships; 11. Recognizing & responding to crisis & safety; 12. Professionalism; 13. Education; 14. Children’s mental health services	Significantly higher knowledge for web-based and in-person training NS difference between in-person and online knowledge gains. Significant differences in employment characteristics between completers and non-completers
Joo (2018) USA	3 (69 peer-client meeting recordings coded & analysed) (100% female) All participants ≥ 50 yrs	Mental health; entry level (service user)	PMC training Current (volunteer) 20 h & 8 wk field training (supervised)	NR	Peer talk focussed on building rapport, emotional support, facilitating talk with clients, & providing information & counselling; Peer self-disclosure used frequently in emotionally responsive talk; Positive global affect consistently high across all meetings; Degree of client-centred talk increased & rapport building decreased over time; Counselling skills used more in first session than later sessions
Olin (2010) USA	15 (NR) *M*_*age*_ = 42.8 (10.3)	Mental health; entry level (carer/family)	Training FPAs as PSWs Current (paid) 10 wkly 4 h sessions & 6 /mo booster session (40 h)	Content: 1. Engagement & community skills; 2. Priority setting & problem-solving skills; 3. Group management skills; 4. Understanding child psychiatric disorders, diagnoses, & treatment; 5. Mental health system; 6. Service options through the education system; 7. Post-training monthly meetings for 6 mos.	Significant impact: 1. Mental health service efficacy; 2. Complex professional skill development (e.g., priority setting & problem solving). NS change in 1. Knowledge; 2. Basic advocacy skills (e.g., engagement, listening, & boundary setting)
Rapp (2008) USA	78 (NR) NR	Mental health; entry level (service user)	PSW service user training Future (paid) 15 wkly 3 h session & 7 wk 104 h intern (149 h)	Content: 1. Helping skills; 2. Theory; 3. Strengths-oriented practice; 4.Recovery & wellness; 5. Cultural competence; 6.Documentation; 7. Confidentiality; 8. Ethics Methods: Active learning; discussions, experiential exercises, reading assignments, role-play; didactic presentations. Monthly support & feedback	Significant increases: 1. Graduate employment & post-secondary education enrolment at all three f/u points; 2. Students working in social services jobs; 3. Percentage of employed students working hrs/wk, with steady growth in number of students working more than 30 hr/wk; 4. Working days of employed graduates
Rodriguez (2011) USA	58 (NR) NR	Mental health; mixed levels of experience/expertise. FPA experience varied" (carer)	Training FPAs Current (NR) 40 h & bi-wkly 1 h consultation calls for 6 mos (88 h)	Content: 1. Conceptual framework; framework of PEP, principles of parent support, behaviour activation; 2. Listening, engagement, & boundary setting; 3. Priority setting, action plan development, & problem solving; 4. Group management; 5. Preparing parents to navigate mental health system; 6. Disorders and treatment; 7. Service options through school system skills Methods: Adult learning approaches; direct instruction to share knowledge or techniques, group support, modelling, vicarious learning, & practice with feedback	Significant increase in family empowerment, mental health services efficacy, and skills post-training, and at f/u. Key FPA activities used: Emotional support and service access issues, especially involving the education system. FPAs reported increase in activities (e.g., role-playing to help develop parent skills)
Stoneking (2007) USA	68 (NR) NR	Mental health; entry level (service user)	PSW training Future (paid) 7 day training & 12 wkly 2 h practicums	Content: Introduction to recovery principles and wellness management; 1. Recovery; 2. Developing a support system; 3. Self-help strategies; 4. Healthy lifestyle; 5. Building self-esteem; 6. Enhancing wellness; 7. Beginning personal journey of recovery; 8. Developing recovery plan Methods: Mindfulness, presentations, homework, role-play & group exercises	Post-training improvements: Knowledge, skills, attitudes. Knowledge and skills emphasized at training improved when applied in work settings after three mos of trainees being employed
Tsai (2017) USA	14 (14.3%) *M*_*age*_ = 45.54 (11.84)	Mental health; entry level (service user)	PSW training NR (NR) 2-day workshop & 2 /mo booster sessions	Content: 1. Understanding mindset in which MI delivered, MI processes (i.e., engage, focus, evoke, plan), & MI components to build an empathic, client-centred relationship; 2. Elicit motivations for change, resolving change ambivalence, & strengthening change commitment Methods: Experiential & interactive learning, live & video demonstrations, booster sessions, group discussion & feedback, coaching	Significant decline in 1. MI inconsistent adherence rates (i.e., reductions in providing unsolicited advice & emphasizing absolute abstinence); 2. Sharing lived experience adherence (i.e., shared common experiences with service recipients less often over time). NS changes in adherence or competence related to MI Fundamental and MI Advance subscales
Wolf (2014) USA	112 (30 telephone survey; 54 mail survey; 28 in-person interviews) (63% female telephone survey; 63% female mail survey; 57% female in-person interviews) Age NR	Mental health; entry level (service user)	PSW training Future (paid) Full-time over 2 semesters	Content: 1. Introduction to mental health systems. 2. Topics in mental health (e.g., conceptual knowledge, clinical & administrative skills) Methods: Course content supplemented by practitioner guest lecturers Practicum in Mental Health – 150-h practical training internship (supervision, written work, seminars)	Recovery/health outcomes: Most reported significant ongoing life challenges. Most employed peers felt good about helping others, had increased self-esteem, made progress in own recovery, increased understanding of own disorder, satisfied with earning income, and positive impact on career Peers vs. non-peers: Education: More non-peers earned a higher degree and were pursuing higher degrees in mental health fields. Employment: Higher among non-peer graduates. Graduates were employed by more than 20 area mental health agencies. High rate of graduates employed in the field and earning degrees or seeking additional higher education (though lower than nonpeers). High employment rates among all graduates, with majority working full-time. Earnings: Comparable % of peers and non-peers earning $30,000–$34,000. More non-peers earning $15,000–$30,000. More peers earning < $14,999
Wolfe (2013) USA	4 (25% female) Age range: 54–69 NR (NR)	AOD; entry level (service user)	PSW training Future (NR) 2 day group sessions, wkly 1.5 h group sessions, wkly 45 min individual sessions) over 4 mos (40 h)	Content: 1. MI spirit (e.g., empathy, respect, & eliciting the point of view of the participant); 2. MI skills (e.g., delivering personal feedback, eliciting, & amplifying ‘change talk,’ asking open-ended questions); 3. Global MI (e.g., evocation, collaboration & autonomy/support, direction and empathy, and identification & reinforcement of change talk) Methods: didactic instruction, group workshops, individual feedback sessions, role play, video demonstrations	Trainees did well in MI styles and strategies assessing/highlighting motivation to change, affirmation and support for change, and change planning. Had difficulty in the authority (telling patient what to do), pros and cons, giving advice, and open-ended questions. MITI ratings: Half of peers achieved treatment fidelity with increases in all global constructs (MI spirit, direction & empathy). All achieved fidelity in MI spirit. (e.g., respectful, non-judgmental client relationship); highlighted client’s perspective; respected client’s decisions

% = Percentage; *AOD* Alcohol and other drugs; *BHP* Behavioural health practitioner; *DSM* Diagnostic Statistical Manual; *F/u* Follow-up; *FPA* Family peer advocate; *HIPAA* Health Insurance Portability and Accountability Act; *Hr* Hour; *Hrs* Hours; *M*_*age*_ Mean age; *Min* Minutes; *Mo* Month; *Mos* Months; /mo = Monthly; *MI* Motivational interviewing; *MITI* Motivational Interviewing Treatment Integrity Code Version 3.0; *NR* Not reported; *NS* Not significant; *PEP* Parent Engagement and Empowerment Program; *PMC* Peer mentor consumer; *PSW* Peer support worker; *QoL* Quality of life; *SS* Seeking safety; *SD* Standard deviation; *T1* Time 1; *T*2 Time 2; *USA* United States of America; *Vs* versus; *Wk* Week; *Wkly* Weekly; *Yr* Year; *Yr* Years

**Table 2:** Sample, study characteristics, training content, and findings from included mixed-methods studies

Study (Year) Country	N (% female) Mean age (SD)	Population; position level (service user/carer)	Training purpose/type (Role)/duration (Total hrs)	Training content	Relevant findings
Atif (2019) Pakistan	45 (100% female) *M*_*age*_ = 30 (5.7)	Mental health; entry level (service user)	PSW training Future (volunteer) 5 day training (30 h)	Content: 1. Psychosocial factors impacting mother & child health during the perinatal period; 2. Counselling skills; 3. Intervention principles, contents & delivery Methods: Lectures, discussions, activities, use of case scenarios, sharing personal experiences, role-play, intervention material & field training	Quan: Most maintained or improved competencies at initial assessment and at f/u. All participants reached satisfactory competency levels. Qual: Training facilitators: 1. Ability to relate to trainers. 2. Perceived usefulness of the training. 3. Training techniques. 4. Linkage with primary health care system. 5. Increased psychosocial awareness and wellbeing Training barriers: 1. Lack of refresher trainings. 2. Household commitments. 3. Fears linked to no prior training exposure
Deren (2012) USA	158 (80 experimental; 78 control) (24% female experimental; 36% female control) *M*_*age*_ = 40.8 (8.8) experimental *M*_*age*_ = 42.3 (8.9) control	AOD; entry level (service user)	POW training Future role (paid) 4–5 day training & 12 wks supervised outreach	Content: 1. Rationale for the project; 2. Overview of HIV and HCV facts; 3. Outreach strategies Methods: Discussions, role-play, supervised outreach in pairs with wkly supervision	1. Most patients completed training. 2. Life crises took priority over training. 3. Many subjective benefits reported. 4. Lower rates of drug use. 5. More likely to talk with others about HIV. 6. More positive about role as Health Educator and higher engagement in additional vocational activities Recommendations: 1. More flexibility in training dates to increase participation and completion. Possibility of rolling admissions and other methods to make up missed sessions. 2. Increased support to those who continue to use drugs
Franke (2010) Australia	50 (survey) 132 (interviews) (NR) NR (NR)	Mental health, Entry- & mid-level (service user)	PSW training Future & current roles (paid) Info session, 6 day training & Community Services Mental Health Cert III course	Content: Peer work roles, boundaries, sharing your story, self-management, & job opportunities Certificate III content NR	Survey: Most (> 90%) found training topics fairly/very useful, most (88%) were very interested in pursuing PSW role and Certificate III course Interviews: Employment (volunteer & paid) and workforce participation increased among course completers over two yrs f/u
Hegedüs (2016) Switzerland	34 (survey – sample 1: *n* = 16; sample 2: *n* = 18) 10 (interviews) (72.2% female sample 1; 75% female sample 2) *M*_*age*_ = 47.5 (7.9) sample 1 *M*_*age*_ = 43.7 (8.9) sample 2 interview gender & *M*_*age*_ NR	Mental health; entry level (service user)	PSW training Future (any capacity) Coursework classes (10 × 3 day sessions held /mo) & 2 practical trainings) over 1 yr	Content: 1. Promoting health & well-being; 2. Trialogue; 3. Empowerment in theory & practice; 4. Experience & participation; 5. Perspectives & experiences of recovery; 6. Independent peer advocacy; 7. Self-exploration; 8. Recovery-based assessment & crisis planning; 9. Peer support; 10. Teaching Methods: 40 h of practical training; 150 h of additional practical training	Employment: At 1-yr f/u, most were employed as PSWs Training satisfaction: Most very satisfied/satisfied with their employment status 1-yr post-training Benefits: Personal and professional development Concerns: 1. Evolving from patient role; 2. Feeling welcome and being confronted with conflicting expectations; 3. Helping others while needing help; 4. Fear of failure
Meehan (2002) Australia	10 (80% female) NR Age range 21–60	Mental health; entry level (service user)	PSW training Future (any capacity) 16 wk (4 wk classroom & 12 wk experiential)	Content: 1. Legal & ethical principles governing inpatient treatment; 2. Mental illness overview; 3. Communication and counselling skills Methods: Lectures, group work, role plays; practical/experiential training working with staff and inpatients at ward level; 4 h per wk for 4 wks in acute, rehabilitation & activities areas. Prior to ward sessions, trainee check-ins. Debriefing post-ward sessions	Qual: Trainees generally satisfied with program format, content, relevance. Contents could include more counselling, patient rights, patient advocacy, & legal issues around regulating patients in hospital. Trainees appreciated interacting with staff and patients on ward—some found this difficult. Trainees recognised difference in relationship between professional staff and patients, and their relationship with patients. Lack of clear job description created problems—Trainees felt insecure when questioned by ward staff about role Quan: State anxiety, trait anxiety, perceived stress, locus of control, & self-esteem either increased or maintained overtime
Tse (2014) Hong Kong	25 (NR) NR	Mental health; entry level (service user)	PSW training Future (paid) 6 wks coursework (10 × 3 h + 1 day workshop) & 24 wk paid internship	Content: 1. Reconstructing own personal recovery; 2. Recovery concept; 3. Peer support concept; 4. Helping skills; 5. Goal setting; 6. Professional codes of conduct; 7. Working relationships; 8. Crisis management; 9. Supervision and self-care On-the-job training: 1. Social worker supervision; 2. Group supervision co-facilitated by social workers and programme consultant every 4–6 wks	Survey responses: *Psychosocial measures*: Trainees scored higher on Recovery and Hope. Self-esteem similar at pre-post. *Training experience*: positive experience of training (i.e., held trainers in high regard & would recommend program). *Overall assessment*: 1. Positive gains—Trainees turned their illness into strength & mood improved. Training prompted them to reach out more to the community and made them realise that they could achieve other things. 2. Factors that helped trainees deal with new role: Support from other trainees, supervisors’ families, & satisfaction from helping. 3. Challenging aspects: Day-to-day tasks seen as challenges e.g., conversing with different people & filling paperwork. 4. Program uniqueness—empathetic & empowering. 5. Expectations – envisioning a future career
Weeks (2006) USA	130 (36.2% female) *M*_*age*_ = 39.8 (7.37)	AOD; entry level (service user)	Trained PSWs Future (NR) 10 sessions (5 × 2 h, 5 field sessions)	Content: 1. Introductions, program concepts & community concerns; risks & solutions role play; 2. HIV/STI/TB risk & prevention; persuasive communication & role play, harm reduction materials, homework; 3. Review PHA intervention; basic hepatitis risk/transmission, model harm reduction, practice contact documentation, role play intervention engagements, identify public advocacy activity; 4. Role play difficult situations; 5. Implement RAP harm reduction/health advocacy intervention in community; return to offices for feedback/sharing Methods: Didactic education; demonstrations; provision of materials; & /mo community advocacy group meetings	Qual: Intervention feasible and appropriate. PHA’s modelled protective behaviours, distributed prevention materials, & encouraged healthier and safer activities. Many hoped their work as PHA could someday become a steady, paying job. Most increased self-worth from helping others despite ongoing struggles; improved own health & well-being; felt more respected by their peers & community members; & improved self-perception; Few had negative experiences; 6. Personal barriers to conducting PHA work Quan: Significant increases in PHA condom use, reductions in sex partners, increases in injectors cooking drug solutions, use of rubber tips by crack-cocaine users, reduction in drug use overall, & increase in PHAs who recently spoke to other drug users about HIV prevention or other health issues and harm reduction. Other harm reduction practices for injection drug users also increased Attitudes toward concept and practice of conducting PHA work significantly improved from intake to post-training

*AOD* Alcohol and other drugs; *Cert* Certificate; *F/u* Follow-up; *HCV* Hepatitis C virus; *HIV* Human immunodeficiency virus; *Hr* Hour; *Hrs* Hours; *IPW* Introduction to peer work; *M*_*age*_ Mean age; *Mo* Month; *NR* Not reported; *NS* Not significant; *PHA* Peer/public health advocate; *POW* Peer outreach worker; *PSW* Peer support worker; *Qual* Qualitative findings; *Quan* Quantitative findings; *RAP* Risk avoidance partnership; *STI* Sexually transmitted infection; *TB* Tuberculosis; *USA* United States of America; *Wk* Week; *Wks* Weeks; *Wkly* Weekly; *Yr* Year

**Table 3:** Sample, study characteristics, training content, and findings from included qualitative studies

Study (Year) Country	N (% female) Mean age (SD)	Population; position level (service user/carer)	Training purpose/type (Role)/duration (Total hrs)	Training content	Relevant findings
Blixen (2015) USA	8 (62.5% female) *M*_*age*_ = 56 (Range: 45–64)	Mental health; entry level (service user)	PSW training Future (NR) 2 days group training & 12 education sessions	Content: Introduction, outline TTIM intervention, communication skills, group leading/co-leading, help-seeking pathways and crisis management, illness self-management including physical & mental health support needs Methods: Role-play; participating in TTIM intervention sessions as facilitators first then co-leaders	Themes: 1. Positive group experience; 2. Success with the training manual; 3. Increased knowledge of mental illness/diabetes; 4. Improved self-management of own mental illness/diabetes; 5. Increased self-confidence; 6. United in purpose
Colon (2010) USA	80 (34% female) *M*_*age*_ = 40.8 (8.8)	AOD; entry level (service user & carer/family)	POW training NR (paid) 5 day training, 12 wks of supervised peer outreach & 2 mo booster sessions	Content & Methods: 1. Training overview; 2: Outreach and HIV/HCV facts; 3: Discussion of outreach strategies and available resources; role plays to practice outreach skills; 4: Further discussion of outreach; preparation of outreach kits for distribution while conducting outreach; 5: Field event: Conducting outreach in the community; discussion of experiences; closing ceremony, certificates of completion; 6. Clinic staff & trainee discussion sharing learnings from training and during outreach	Benefits: Improvement in pertinent knowledge, self-efficacy for working in a community navigation role. Harm reduction approach perceived to increase retention of trainees in program Challenges: Attending training under influence of drugs and attending all training sessions Limitations: A need to assist trainees to transition to other peer educator roles Recommendations: Flexibility in training offerings; need for post-training support; counselling support for dealing with stressors of the peer outreach role and work through ongoing drug and mental health issues; support interpersonal issues and help trainees utilise and develop skills to transition to other roles post training
Gerry (2011) UK	17 (NR) NR	Mental health; entry level (service user)	PSW training Future role (NR) 2 wk training	Content: 1. Recovery; 2. Power of peer support; 3. Self-esteem and self-talk; 4. Meaning & purpose; 5. Telling your personal story; 6. Communication; 7. Employment as a path to recovery; 8. Being with people in challenging situations; 9. Peer support in action	Benefits: Increased confidence & capacity of inter- & intrapersonal skills; personal growth, increased self-esteem & confidence, feeling empowered & hopeful, improved life quality Limitations: Training exhausting & intense Recommendations: Run training over a longer time period Post-training challenges: Lack of involvement in professional growth impeded initial attainment of career goals; trainees perceived the trust's uptake of the recovery approach to be 'tokenistic'
King (2009) Australia	12 (58.3% female) *M*_*age*_ = 37.5 (13.3)	Mental health; entry level (service user)	PSW training Current (volunteer) NR	NR	Themes reported in terms of frequency of appearance in transcripts; Typical (T; 6–7); General (G; 4–6); Variant (V; 2–4) Domain 1: 1. *‘Experience of the training’*: Found training beneficial (T); prepared using own past experience/program resources (T); inadequately prepared (V). 2. ‘*Experience of supports available/utilised’*: Fellow peer outreach volunteers as supports (T); program staff as supports (T). Need more formal support (V); Need for training in specific skills (V) Domain 2: 1. ‘*Perceived benefits to self’*: Skill improvement (G); personal reward from helping others (T); greater confidence & self-worth (T); ability to relate to people (V); insight (V). 2. ‘*Challenging aspects of outreach work’*: being reminded of past relapses (T); talking with outreach recipients who are very unwell or uninterested (T); lack of status & skill compared to healthcare professionals (V); taking issues of outreach home (V); difficulty communicating/managing own psychiatric disability (V)
Sanchez-Moscona (2021) Spain	16 (NR) NR	Mental health; entry level (service user)	PSW training Future role (NR) NR	Content: 1. Pedagogy applied to peer support training & to recovery, group dynamics; 2. Basic concepts of peer support, accompaniment & mutual aid groups, rights, language & communication, risks & limits; 3. Mental health system agenda & comparison of training models Methods: Participatory methods, role-playing, debate, & discussion	Benefits: Learning theories, teamwork, practical exercises. Content appropriate & taught respectfully, encouraging learning. Trainees valued understanding, confidence, skills, & knowledge acquired. Through practical exercises, trainees developed critical reasoning & joint learning construction. Learning objectives for each session achieved by most Limitations: Theory heavy, little practical content, training intense as large amount of information. Suggested more general training elements added
Simpson (2014) UK	13 (30.8% female) *M*_*age*_ = 42 (6.71)	Mental health; entry level (service user)	PSW training Future (any capacity) 12 wkly × 6 h sessions (72 h)	Content: 1. Exploring PSW; 2. Tree of life; 3. Recovery & personal recovery plans; 4. Confidentiality, information sharing, boundaries; 5. Active listening; 6. Social inclusion; 7. Appreciating differences; 8. Responding to distressing situations; 9. Revisiting boundaries & difficult situations; 10. PSW preparation; 11. Ending Methods: Narrative sharing; group work; role-play; discussions; post-training support & supervision	No change in trainee experiences & feelings. Training provided good PSW preparation (e.g., confidence, pride) Benefits: Supervision, role-plays, support groups Limitations: Could not cover all content, insufficient preparation for emotional reaction of the work, no family-specific training, desire for more practical training
Stockmann (2019) UK	56 (NR) NR	Mental health; multiple professions & positions represented (service user)	PSW training Current role (NR) 4 wkly sessions over 1 yr	Content: 1. Introduction, family therapy, OD & self-work; 2. Deepening OD practice & self-work; trauma-informed & recovery-based approaches to mental health care; 3. Applied OD practice, peer support & integration; 4. Holistic approaches; reflections, & final assessments Methods: Experiential exercises, practice in reflective processes, self-disclosure tasks, family-of-origin activities, role-play, lectures, yoga, mindfulness, online platform	Benefits: 1. Enhanced personal & professional development; 2. Training principles that worked well: Mindfulness & value of clinician/patient perspective Limitations: 1. Balancing power within teams; 2. Early training uncertainty, insufficient explanations & feedback; 3. Directive teaching anxiety provoking; 4. Preferred more instruction, more experiential or reflective exercises; 5. Lack of feedback; 5. Discussion platform caused frustration
Stewart (2008) Australia	35 (54.3% female) NR	Mental health; entry level (service user)	NR Current (any capacity) NR	NR	Future training needs: Advocacy, administration, policy & legislation, management, counselling/therapy, staff development, meeting skills, skill development (e.g., conflict resolution & aggression management, confidence building, stress management, assertiveness); understanding health system & associated jargon Most support mandatory training
Toikko (2016) Finland	12 (NR) NR	Mental health; entry level (service user)	PSW training Future (paid) 1 day fortnightly over 10 mos	Content: 1. Personal experiences with mental health; 2. Mental health-produced knowledge from a professional perspective; 3. Expertise of health & social service users Methods: Interactive groups	Themes: 1. Creating distance from experiences; 2. Sharing experiences; 3. Combining experiences with competences; 4. Developing future orientation. Training produced new activities within hospital in which experts by experience took part. Participants who had active roles within the hospital extremely satisfied with training & tasks since training
Treloar (2012) Australia	18 (61.1% female) NR (Age range: 27–54)	AOD; entry level (service user)	PSW training Future (paid) 11 sessions	Content: 1. Hygiene & injecting practice in HCV prevention; 2. Risk related to routine and habit in injecting practice Methods: Videos, group discussions, development of peer education messages, & strategies	1. Participants identified strategies influenced by macro- (social & legal contexts) & meso-levels (organizational & funding) that could be employed; 2. Gathered knowledge about safer injecting & HCV prevention; 3. Acknowledged own & peers’ experiences (e.g., realities of social & economic marginalization) 4. Highlighted need for flexible programs & supportive funders
Willging (2016) USA	4 (NR) NR	Mental health & AOD; entry level (service user)	CPA training Future (paid) 4 day training & coaching	Content (in-person training): Mental health & substance abuse, minority stress, diversity within LGBTQ communities, & rural treatment systems; helping skills, support for people seeking mental health services; skills on needs assessment, solution-focused helping, suicide prevention, conducting presentations, negotiating communication conflicts, outreach, ethical decision making, & self-care Content (phone coaching): Ongoing mentorship Content (phone, online): Forums	Themes: 1. Coaching support – Coaching support appreciated, although, full-time coordinator & supervisor would strengthen & improve access to in-depth consultation. 2. Skills & preparation – Advocates felt unprepared & unsupported to perform outreach given truncated timeline. Additional training required. 3. Working with help seekers – Perception of work as significant & valuable; confidence in new roles; learnt value of their work & applicability to case management; offered supportive relationships without judgment; provided optimal support for less-distressed help seekers, although felt underprepared when working with severe/complex cases; experienced delays or challenges in connecting & maintaining contact with help seekers; required support with decision making & protecting their boundaries. 4. Negotiating diversity – Increased familiarity, knowledge, & confidence with different sexual identities. Additional support needed for work with culturally & socio-economically diverse populations. 5. Logistical challenges in rural contexts. 6. Systemic challenges in navigating the mental health system & models of care

*AOD* Alcohol and other drugs; *CPA* Consumer peer advocate; *HCV* Hepatitis C virus; *HIV* Human immunodeficiency virus; *Hrs* Hours; *LGBTQ* Lesbian, gay, bisexual, transgender, and queer; *NR* Not reported; *M*_*age*_ Mean age; *Mo* Month; *Mos* Months; *OD* Open dialogue; *POW* Peer outreach worker; *PSW* Peer support worker; *TTIM* Targeted training in illness management; *UK* United Kingdom; *USA* United States of America; *Wk* Week; *Wks* Weeks; *Wkly* Weekly; *Yr* Year

### Quality Assessment and Risk of Bias

The Quality Assessment for Diverse Studies tool (QuADS, Harrison et al., 2021) was used to appraise quality and risk of bias of included references. The QuADS is suitable for systematic reviews of mixed- or multi-method studies and different study designs, consisting of 13 items scored on a four-point scale (0 = not reported, 1 = reported but inadequate, 2 = reported and partially adequate, 3 = sufficiently reported). An additional item from the Jadad Scale for Reporting Randomised Control Trials (Jadad et al., 1996) was included to assess for study randomization where relevant. This item was scored on a three-point scale (0 = not reported, 1 = described as randomised but method not described or inappropriate, 2 = described as randomised with appropriate method of randomisation used). This resulted in four risk-of-bias levels for items 1–13 (i.e., low, low-moderate, moderate-high, & high), and three levels of risk (i.e., low, moderate, & high) for item 14. Per study, the maximum quality assessment score that could be achieved was 41; with the Jadad item excluded, a maximum score of 39 could be achieved.

## Results

### Study Selection

The database search revealed 2724 records. After duplicates were removed, 2432 studies were screened for eligibility. The title and abstract screen excluded 2339 studies and a further 57 were excluded following the full-text screen. Thirty-six papers met eligibility criteria for inclusion. Study sample, characteristics, training content, and relevant findings of each are provided in Tables 1–3.

### Characteristics of Included Studies

Half (*n* = 18) of the 36 included studies used quantitative methods, 10 were qualitative, and eight studies used a mixed method design. Publication year ranged from 2000 to 2021. Most studies were conducted in the United States (*n* = 21), with six from Australia, three from the United Kingdom, and two from Switzerland. Countries from which one study was yielded were Pakistan, Spain, Finland, and Hong Kong.

**Fig. 1:**
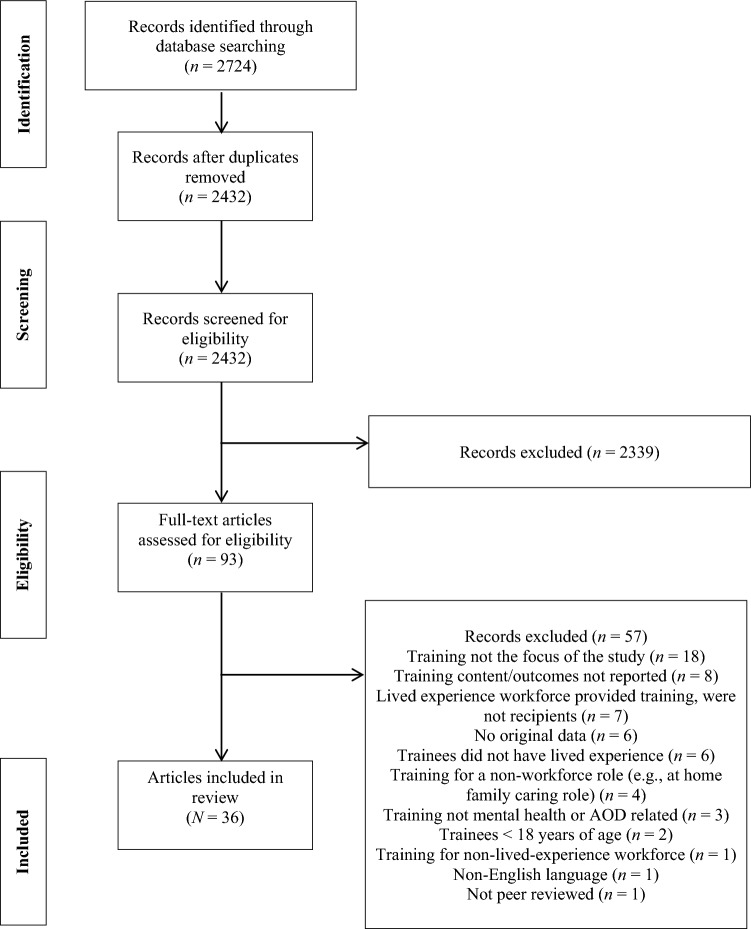
PRISMA diagram of the phases of the review process and record selection

Most studies (*n* = 28) reported on training programs for service users, four were for carers, and one addressed training for both service users and family carers. Three did not indicate the specific LLE target population for the training. In terms of training setting, most were mental health (*n* = 28) specific. Five programs related to the AOD population, two programs were for mental health or AOD, and one study required participants to have a dual diagnosis (substance use in those with mental illness). Training in more than half of all studies (*n* = 20) was for future roles, nine were for current roles, two training programs were for both future and current roles, and five studies did not report this information. Most training programs (*n* = 29) were tailored specifically for those looking for entry level positions. Three programs were specific for entry and/or mid-level positions, with no studies examining training for experienced workers. Four programs were vague/did not report this information. Most studies were designed for those interested in either paid or unpaid future roles. Training length was highly variable ranging from a focused workshop of 5 h to a spread of training activities spanning 1.5 years. Most training (*n* = 26) included solely coursework, while 10 programs included course work and a practical training component. Seven programs provided specific post-course follow-up supervision. See Fig. 1 for a PRISMA diagram outlining the identification, screening, eligibility, and inclusion process of examined literature.

### Quality Assessment

**Fig. 2:**
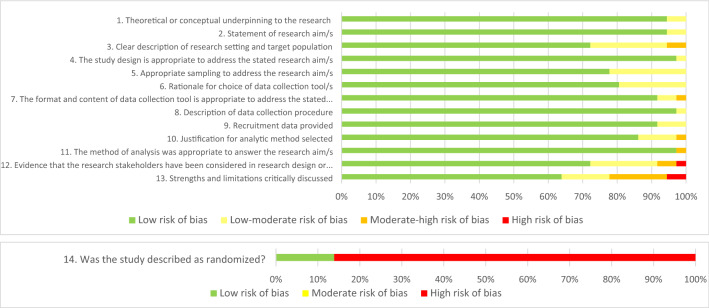
Quality assessment graph. Reviewer’s judgements regarding each risk of bias item, as presented as percentages for the 36 included studies using a modified version of the Quality Assessment for Diverse Studies (QuADS) tool. For items 1–13, a score was assigned for each criterion on the checklist using a 4-point rating scale developed by Harrison et al. (2021). A score of 0 reflects = not reported; 1 point = reported but inadequate; 2 points = reported and partially adequate; 3points = denotes a low risk of bias sufficiently reported and adequate. An additional item (item 14) was further included from the Jadad (1996) measure to assess for a randomization using 3-point rating scale was used. A score of 0 = not reported; 1 item described as randomised but method not described or inappropriate; 2 = described as randomized with appropriate method of randomization used. The 14-item modified QuADS was not used as a means of study exclusion, but rather as an indicator of study quality across included studies

Quality of included studies was high, averaging 36.92 out of a total score 41 on the Quality Assessment for Diverse Studies tool (QuADS; Harrison et al., 2021) and the Jadad (1996) measure. Irrespective of study quality, it was determined apriori that no study would be excluded based on quality or risk of bias. The highest scoring item was item 8 (Data collection procedure) which was sufficiently reported by 97.22% (*n* = 35) of studies, followed by items 1 (Theoretical and conceptual underpinning of the research) and 2 (Research aim/s) which were both sufficiently reported by 94.44% of studies (*n* = 34). The lowest scoring item was item 14 (Study described as randomised), sufficiently reported by only 13.89% of studies (*n* = 5), followed by item 13 (Strengths and limitations critically discussed) which was sufficiently reported by 63.89% of studies (*n* = 23). Quality assessment results for individual items are graphically presented in Fig. 2. See Online Appendix 3 for Quality assessment table.

### Training Program Design, Delivery, Content, and Method

Many studies (*n* = 18) did not report on who developed the training content that was examined. Majority of studies (*n* = 19) did not specify whether the research was led and/or co-produced by LLE researchers. Six of the 36 (16.7%) training programs were designed by service users alongside a clinician or researcher, or through service user consultation. Two training programs were designed solely by service users (Franke et al., 2010; Tse et al., 2014), one was developed by peer support coordinators (Simpson et al., 2014), and one was developed by community partners (Compton et al., 2014). Mental health professionals designed five training programs, while researchers designed three programs. A substantial proportion of studies did not report who delivered the training (*n* = 13). For those that did report this information (*n* = 23), 14 were delivered by a mental health professional or employed trainers, one by researchers, two by peers, five by a combination of peers (service user or advocates) and health professionals, and one by mental health advocates only. Out of the 36 training programs, only three (8.3%) programs (Stoneking & McGuffin, 2007; Treloar et al., 2012; Tse et al., 2014) were service user co-designed and co-delivered.

**Fig. 3:**
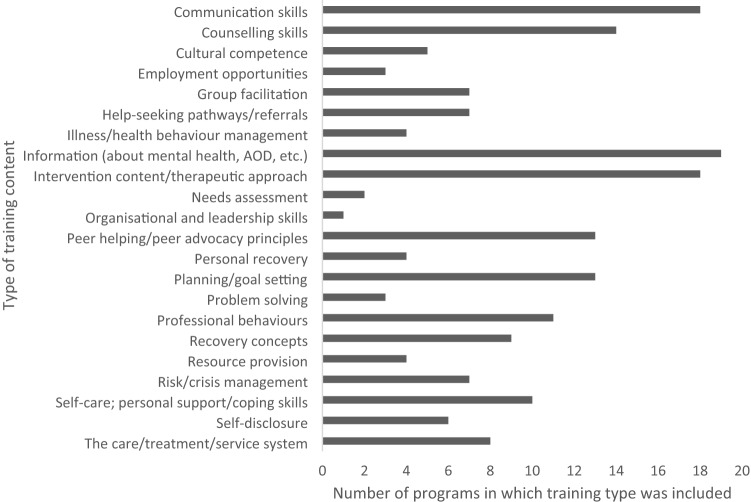
Type of content included in training programs. Personal recovery refers to content regarding the trainees’ own personal recovery journey. Recovery concepts relate to principles or foundations of recovery as applied to work with persons receiving mental health or AOD services

The content included in the training programs was described in 33 of the 36 included papers and is presented in Tables 1, 2 and 3. Content is summarised into categories and shown in Fig. 3. The most commonly included elements in training were information (e.g., about mental health or AOD, related factors and context; *n* = 19), content addressing the intervention/practice concepts (*n* = 18), and communication skills (*n* = 18). Counselling skills (*n* = 14), peer helping or peer advocacy concepts (*n* = 13), planning and goal setting (*n* = 13) and professional concepts such as confidentiality, boundaries, and client rights (*n* = 11) were also included across numerous training programs. Other content infrequently involved in training included organizational and leadership skills (*n* = 1), needs assessment (*n* = 2), employment opportunities (*n* = 3).

**Fig. 4:**
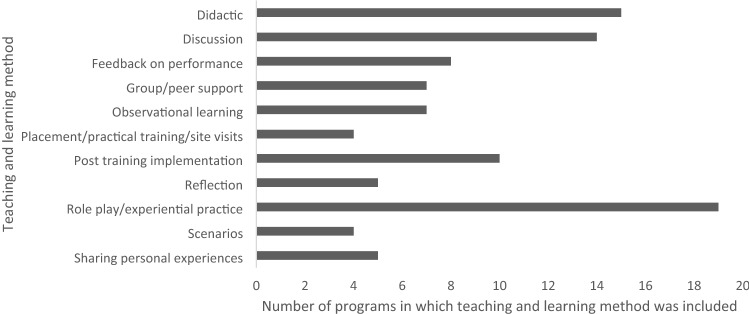
Methods of teaching and learning in delivery of training programs

Methods to deliver training were described by 28 of the 36 papers (see Tables 1–3). The categories of training methods, collated from descriptions of training programs, are shown in Fig. 4. Role-play and experiential practice (*n* = 19), didactic (*n* = 15), and discussion (*n* = 14) methods were most commonly used. Several training programs also described the use of post-training implementation of the intervention, either co-delivered with an experienced person or with supervision, as part of the training program (*n* = 11).

Twenty-five of the training programs were delivered in-person, two were blended modes (Stockmann et al., 2019; Willging et al., 2016), and one study looked at both variants—an online-based training program compared with an in-person training program (Horwitz et al., 2020). Eight papers did not indicate the delivery mode.

### Findings from Training Participation

Studies investigating LLE workforce training programs examined a range of outcomes and factors associated with participation in training. Ten outcome themes were identified. The most frequently examined outcomes related to professional development (*n* = 28), training limitations (*n* = 17), personal development (*n* = 17), and training satisfaction/utility (*n* = 16). Other outcomes included application of training skills to the work role (*n* = 12), post-training employment (*n* = 7), trainee reservations (*n* = 7), personal barriers to training participation/completion (*n* = 7), post-training education (*n* = 6), and mental health service-self efficacy (*n* = 4). These outcomes are described below. Thirteen studies reported on pre-to-post changes in outcomes or provided evaluations at post-test alone. Outcomes in 12 studies were assessed at multiple time points (pre-test, post-test, mid-test, and/or follow-up). Four studies assessed outcomes during the training implementation, and another four assessed pre- to follow-up change in outcomes, or at follow-up alone.

Quantitative research methods were frequently employed when assessing post-training employment (*n* = 7, 100%), post-training education (*n* = 6, 100%), mental health service self-efficacy (*n* = 4, 100%), training applicability (*n* = 7, 58%), and professional development outcomes (gains in skills and knowledge; *n* = 15, 54%). Qualitative research methods were commonly implemented when examining trainee reservations (*n* = 7, 100%), training limitations (*n* = 15, 88%), personal barriers to training (*n* = 6, 86%), trainee satisfaction (*n* = 12, 75%), and personal development (*n* = 10, 59%).

#### Professional Development

Nine studies reported general improvements in knowledge and skills from participating in training, immediately post-training (Bentley, 2000; Cleary et al., 2009; Compton et al., 2014; Hoagwood et al., 2018; Horwitz et al., 2020; Sanchez-Moscona & Eiroa-Orosa, 2021; Simpson et al., 2014) and at follow-up (Hegedüs et al., 2016; King et al., 2009). Across ten studies, trainees developed program-specific skills during the course of training participation (Blixen et al., 2015; Treloar et al., 2012; Willging et al., 2016), immediately after training (Crisanti et al., 2016; Gerry et al., 2011; Joo et al., 2018; Meehan et al., 2002; Weeks et al., 2006) and at follow-up (Rodriguez et al., 2011). Specifically, trainees demonstrated gains in skills relating to mental health knowledge (Blixen et al., 2015), prevention practices (Joo et al., 2018; Treloar et al., 2012; Weeks et al., 2006), counselling ability (Crisanti et al., 2016), communication/collaborative skills (Gerry et al., 2011; Joo et al., 2018; Meehan et al., 2002; Rodriguez et al., 2011), and confidence/familiarity with diverse populations (Willging et al., 2016). Three studies reported improvements in attitudes and beliefs via self-report measures immediately after training completion (Cleary et al., 2009; Stoneking & McGuffin, 2007; Weeks et al., 2006). In one study, training did not significantly impact change in knowledge but did impact perceptions of overall professional skills (i.e., complex skill areas) (Olin et al., 2010). In another pre-post study design, trainees gained an understanding of motivational interviewing (MI) styles and strategies, particularly highlighting the motivation to change, but did not perform well on the skills thought necessary to prompt change (Wolfe et al., 2013). Five studies found that trainees had developed a positive orientation towards their careers at 5.3 months (Deren et al., 2012) to one year follow-up (Hegedüs et al., 2016; Toikko, 2016), and immediately post-training wherein course length ranged from 6 weeks to over a 12 month period (Stockmann et al., 2019; Tse et al., 2014).

In terms of competency, training increased or maintained competencies for the vast majority of trainees two years after training, based on self-report assessment (Atif et al., 2019). A qualitative study found that most respondents were in favour of the need for ‘competency’ in various skills to fulfil their roles (Stewart et al., 2008). For two studies, training led to some pre-to-post and follow-up changes in MI competence and practices (Tsai et al., 2017; Wolfe et al., 2013). Trainees also found it helpful to combine their personal experiences with existing professional competencies (Toikko, 2016).

#### Application of Training Skills

Over time (from baseline to follow-up periods), trainees maintained or increased their use of various skills emphasized during training, such as counselling, communication skills, reflective listening, open questions, eye-contact and problem-solving (Bentley, 2000). Trainees also demonstrated pre-to-post training improvements in advocacy skills related to enquiring about resources (Gammonley & Luken, 2001), client-centred talk, positive rapport building and emotional support (Joo et al., 2018), priority setting, role-playing to support parents with service access (Rodriguez et al., 2011), and knowledge and skills important in the recovery process (Stoneking & McGuffin, 2007). One year following training, trainees reported increased confidence in talking with others about the subject matter (Deren et al., 2012), encouraging sharing of experiences in informal, everyday situations (Toikko, 2016), and making use of their own past experience/program resources in the workplace two-weeks following training (King et al., 2009). Midway through training implementation, trainees reported increased confidence in performing outreach (i.e., providing supportive relationships; Willging et al., 2016). Across three studies, trainees indicated that the training had prepared them for future roles as a peer support worker (Cronise et al., 2016; Franke et al., 2010; Simpson et al., 2014). Three studies found an increase in mental health service self-efficacy (the extent to which family peer advocates felt confident in their ability to assist families access mental health services Hoagwood et al., 2018; Olin et al., 2010; Rodriguez et al., 2011) while one found an increase in self-efficacy within a community navigation role (Compton et al., 2014).

#### Post-training Employment

Of the seven papers that addressed employment outcomes, six indicated an increase in employment rates (in any capacity) related to the mental health system at both follow-up (Franke et al., 2010; Gammonley & Luken, 2001; Hegedüs et al., 2016) and post-training periods; Hegedüs et al., 2021; Rapp et al., 2008; Wolf, 2014). One found that more completers were employed in a related field or in accredited settings compared to non-completers (Horwitz et al., 2020). In one study, workload capacities improved with a steady growth in the number of graduates who worked over 30 h per week post-training (Rapp et al., 2008). Another study revealed that the majority of graduates were employed in full-time capacities, although LLE graduates were highly employed in part-time capacities compared to non-LLE graduates (Wolf, 2014).

#### Post-training Education

Among the six studies that evaluated education outcomes, two found increased educational involvement six-months to two years post training (Gammonley & Luken, 2001; Rapp et al., 2008), two found increased planning to pursue further education (Cronise et al., 2016; Franke et al., 2010), and one found that those who participated in outreach work as part of training engaged in extra vocational activities during the follow-up period (Deren et al., 2012). Though at lower rates than non-LLE trainees, one study highlighted that LLE trainees were seeking additional higher education as well as obtaining degrees in mental health fields (Wolf, 2014).

#### Personal Development

Eight studies reported improved perception of self (e.g., self-esteem and confidence; Blixen et al., 2015; Gerry et al., 2011; King et al., 2009; Meehan et al., 2002; Simpson et al., 2014; Wolf, 2014)*,* self-efficacy (Hegedüs et al., 2021), and improved self-image (Weeks et al., 2006)*.* Six studies reported short to long-term improvements in health-related measures (i.e., improved psychosocial wellbeing and quality of life) following training (Atif et al., 2019; Gammonley & Luken, 2001; Gerry et al., 2011; Joo et al., 2018; Stockmann et al., 2019; Weeks et al., 2006). Two studies highlighted a boost in trainees’ relationships with their peers and colleagues (Stockmann et al., 2019; Tse et al., 2014), and one study reported an increase in perceived respect received from others (Weeks et al., 2006). Trainees felt a sense of empowerment as reported in three studies (Gerry et al., 2011; Rodriguez et al., 2011; Willging et al., 2016). In eight studies, trainees gained insight into their own recovery (Blixen et al., 2015; Hegedüs et al., 2016, 2021; King et al., 2009; Simpson et al., 2014; Toikko, 2016; Tse et al., 2014; Wolf, 2014) and in five studies, experienced personal gains from helping others (King et al., 2009; Tse et al., 2014; Weeks et al., 2006; Willging et al., 2016; Wolf, 2014). In four studies, some psychological wellbeing measures such as mental health, quality of life, anxiety or stress, locus of control and self-esteem remained stable or improved but this pre-to-post change was not significant (Hegedüs et al., 2021; Joo et al., 2018; Meehan et al., 2002; Tse et al., 2014).

#### Training Satisfaction/Utility

In five training programs in which it was assessed, the trainings were regarded as useful or beneficial by participants (Atif et al., 2019; Cleary et al., 2009; Deren et al., 2012; Franke et al., 2010; King et al., 2009), and content was appropriate and useful (Bentley, 2000; Franke et al., 2010; Meehan et al., 2002; Sanchez-Moscona & Eiroa-Orosa, 2021; Stockmann et al., 2019). In seven studies, support from other trainees and coaches/supervisors (Colon et al., 2010; King et al., 2009; Simpson et al., 2014; Stockmann et al., 2019; Tse et al., 2014; Willging et al., 2016), and family and service users (Tse et al., 2014) were seen as motivating factors. In over half of the studies that addressed training satisfaction, trainees indicated that they valued the way the course was run such as the style of teaching (Bentley, 2000), training methods/techniques (Atif et al., 2019; Bentley, 2000; Simpson et al., 2014), sharing of experiences (Bentley, 2000), joint learning space/positive group experiences (Blixen et al., 2015; Sanchez-Moscona & Eiroa-Orosa, 2021; Simpson et al., 2014; Stockmann et al., 2019; Toikko, 2016), online discussion platform (Stockmann et al., 2019), empathic/welcoming environment and empowering experience (Tse et al., 2014), and training being well-organized and interesting (Cleary et al., 2009). In one study, payment (stipend) provided incentive to attend, and the harm reduction approach was perceived to increase retention of trainees in program (Colon et al., 2010). In two studies, trainees were highly satisfied with the training provided (Cleary et al., 2009; Crisanti et al., 2016). In one study, most were in favour of mandatory training (Stewart et al., 2008). Additionally, trainees in two studies recommended the training program to others (Cleary et al., 2009; Tse et al., 2014).

#### Training Limitations

Following training, four studies found that additional support was required to help trainees deal with ongoing interpersonal and drug/mental health issues (Colon et al., 2010; Deren et al., 2012; Hegedüs et al., 2016), or life challenges (Wolf, 2014). Further support was also recommended to promote professional growth immediately post-training (Gerry et al., 2011) and at follow-up (Colon et al., 2010), and some indicated the requirement for more formal support (e.g., coaching, supervisionKing et al., 2009; Willging et al., 2016). Two training programs were seen as too condensed and emotionally intense—trainees recommended that training be spread out over a longer time frame (Gerry et al., 2011; Sanchez-Moscona & Eiroa-Orosa, 2021).

Across four studies, respondents thought that the training did not cover or explain topics in great depth (Stockmann et al., 2019; Willging et al., 2016) or suggested more aspects to be covered in the course content (Meehan et al., 2002; Sanchez-Moscona & Eiroa-Orosa, 2021), although trainees in one study acknowledged that it is not possible to cover everything (Simpson et al., 2014). Respondents across six studies reported feeling inadequately prepared for application of specific skills, particularly in relation to advocacy or outreach work/when directly engaging with clients, families, or help seekers (Franke et al., 2010; King et al., 2009; Olin et al., 2010; Simpson et al., 2014; Stewart et al., 2008; Willging et al., 2016). Trainees in two studies encountered difficulties when interacting with peers/families (Meehan et al., 2002; Willging et al., 2016), while some trainees in one study faced resistance or negative reactions from others they approached (Weeks et al., 2006). Trainees in two studies also felt that there was too much emphasis on theory and were in need of more hands-on training (Sanchez-Moscona & Eiroa-Orosa, 2021; Simpson et al., 2014).

Other training issues included learning challenges such as preference for more instructive trainers and experiential or reflective exercises, or training lacking feedback (Stockmann et al., 2019), lack of refresher trainings (Atif et al., 2019), and trainees being opposed to trainer’s ‘tokenistic’ recovery approach (Gerry et al., 2011). Trainees were also challenged with balancing power within teams (Stockmann et al., 2019). One study reported no further details beyond simply suggesting more training in the future (Bentley, 2000), although trainees in another study reported that mandating training could act as a barrier to service user participation (Stewart et al., 2008).

#### Trainee Reservations

Two studies reported that trainees experienced uncertainty and reservations at the start of training (Atif et al., 2019; Stockmann et al., 2019). Across five studies, trainees also expressed concerns in their direct work with peers – they felt unprepared and unsupported (Willging et al., 2016), overwhelmed by tasks (Tse et al., 2014), insecure when questioned by staff (Meehan et al., 2002), confronted by conflicting expectations and were afraid they would fail at fulfilling their roles (Hegedüs et al., 2016). Some trainees sensed that they lacked the status and skills of healthcare professionals (King et al., 2009).

#### Personal Barriers to Training Participation/Completion

Barriers to involvement included household commitments (Atif et al., 2019), scheduling conflicts, and other personal/family crises that interfered with participation (Colon et al., 2010). To maximise participation and cater for the needs of trainees, three studies identified a need for flexibility in the training offerings (Colon et al., 2010; Deren et al., 2012; Treloar et al., 2012). In one study, dropout rates were especially common among men with psychiatric disability (Cunningham et al., 2020). Three studies described personal barriers while engaging in peer outreach and advocacy work, including barriers as a result of current involvement with drugs (Deren et al., 2012; Weeks et al., 2006), homelessness, problems with the police (Weeks et al., 2006), and current difficulty communicating/managing their own psychiatric disability (King et al., 2009).

### Training Content and Outcomes for Trainees

To explore impact of participation in training on trainee outcomes, we summarized outcomes according to included training content. Training programs that included content on both information and intervention/practice concepts were associated with multiple positive outcomes, including enhanced professional development (e.g., knowledge and skills, Atif et al., 2019; Cleary et al., 2009; Crisanti et al., 2016; Hoagwood et al., 2018; Rodriguez et al., 2011; Stockmann et al., 2019; Weeks et al., 2006), personal development (Weeks et al., 2006; Wolf, 2014), training satisfaction/utility (Cleary et al., 2009; Crisanti et al., 2016; Deren et al., 2012; Stockmann et al., 2019; Weeks et al., 2006), and training applicability (Deren et al., 2012; Gammonley & Luken, 2001; Rodriguez et al., 2011). For training programs that did not include content on information or intervention/practice concepts, trainees reported difficulties directly interacting with peers (Franke et al., 2010; Sanchez-Moscona & Eiroa-Orosa, 2021; Tse et al., 2014), were unsure how to assist family members (Simpson et al., 2014), or were in need of more practical opportunities to apply their skills (Sanchez-Moscona & Eiroa-Orosa, 2021; Simpson et al., 2014).

Peer helping/peer advocacy principles were common content components and associated research found increases in employment involvement (Franke et al., 2010; Gammonley & Luken, 2001; Hegedüs et al., 2016; Horwitz et al., 2020; Wolf, 2014) and personal development (Gammonley & Luken, 2001; Gerry et al., 2011; Hegedüs et al., 2016; Simpson et al., 2014; Stockmann et al., 2019; Toikko, 2016; Tse et al., 2014; Wolf, 2014). Content that included both peer helping/peer advocacy principles and professional behaviours (i.e., confidentiality, boundaries, code of conduct, client rights) was associated with enhanced professional development (knowledge and skillsHorwitz et al., 2020; Sanchez-Moscona & Eiroa-Orosa, 2021; Simpson et al., 2014; Tse et al., 2014) and training satisfaction (Cronise et al., 2016; Franke et al., 2010; Sanchez-Moscona & Eiroa-Orosa, 2021; Simpson et al., 2014; Tse et al., 2014). However, where both of these components were absent from training, trainees reported struggling to effectively connect and engage with their peers whilst performing outreach or advocacy work (Olin et al., 2010; Weeks et al., 2006; Willging et al., 2016). Further, where training did not include content on peer helping/peer advocacy principles, trainees felt underprepared when dealing with people with severe mental health and substance use issues or ill-equipped to handle diverse clients (King et al., 2009; Meehan et al., 2002; Tse et al., 2014; Willging et al., 2016). In addition, where content on professional behaviours was not included in training, trainees indicated that more attention was needed to help with professional skills, such decision making and protecting personal boundaries (Olin et al., 2010; Willging et al., 2016).

Where training content included communication skills, frequently observed outcomes included enhanced personal development (Gammonley & Luken, 2001; Gerry et al., 2011; Hegedüs et al., 2016; Meehan et al., 2002; Rodriguez et al., 2011; Weeks et al., 2006; Willging et al., 2016), and perceptions that training content had high workplace applicability/translational value (Bentley, 2000; Gammonley & Luken, 2001; Rodriguez et al., 2011; Willging et al., 2016). Moreover, outcomes from studies where training content included both communication skills and counselling skills, gains in professional development (knowledge and skills) were observed (Bentley, 2000; Compton et al., 2014; Hoagwood et al., 2018; Meehan et al., 2002; Willging et al., 2016; Wolfe et al., 2013) as were higher rates of trainee program satisfaction (Bentley, 2000; Cronise et al., 2016; Meehan et al., 2002; Willging et al., 2016). With the absence of both or either of these training elements, some trainees felt unprepared to engage with pre-existing workplace staff as they lacked confidence and skill in 1) conversing with staff members; and 2) working collaboratively with them and clients (Franke et al., 2010; Olin et al., 2010; Simpson et al., 2014; Tse et al., 2014; Weeks et al., 2006).

Enhanced personal development outcomes were observed following training which included content on recovery concepts (Gerry et al., 2011; Stockmann et al., 2019; Tse et al., 2014), self-care; personal coping skills (Tse et al., 2014; Willging et al., 2016; Wolf, 2014), personal recovery (Gerry et al., 2011; Simpson et al., 2014; Tse et al., 2014), and illness/health behaviour (self-) management (Blixen et al., 2015; Weeks et al., 2006). Where this training content was not included in training programs, trainees reported struggling to manage their ongoing drug/mental health issues (Colon et al., 2010; Deren et al., 2012; Hegedüs et al., 2016). Furthermore, training content relating to self-disclosure appeared to be associated with outcomes of improved peer support interactions and meaningful and constructive use of their lived experience in their workplace (Stockmann et al., 2019; Toikko, 2016). In the absence of self-disclosure content, trainees reported struggling to effectively engage (Meehan et al., 2002; Olin et al., 2010; Simpson et al., 2014), connect with their peers (Willging et al., 2016), and required more guidance on how to meaningfully share their common experiences to motivate others in their recovery efforts (Tsai et al., 2017).

## Discussion

Findings indicate short- and long-term impacts of training participation for this emerging workforce, with the most promising outcomes being increased professional knowledge and skills and improved personal psychosocial wellbeing and trauma recovery. Other positive training outcomes included high trainee satisfaction, increased application of training skills, and employment/education opportunities following training completion. Gaps and training limitations pertained to the training content/delivery, trainee reservations, and personal barriers to training participation or completion. The positive outcomes and their implications elucidated in this study are a much-needed addition to the scarcity of evidence for the utility of LLE training programs. We hope this evidence will further support the professionalisation and standardisation of training in the LLE workforces.

While there is little prior research in relation to LLE training and associated outcomes, results of the present study appear consistent with this emerging evidence base. Specifically, previous research has highlighted that sufficient LLE worker training and support is associated with job satisfaction. In particular, in a study of lived experience workers in Switzerland, Burr et al. (2020) demonstrated that sufficient worker training, specifically in the work areas focused on by the role, resulted in greater job satisfaction. Further to this finding, Scanlan et al. (2020) found that strong job satisfaction for LLE workers was significantly protective against job disengagement, burnout, and turnover.

Training limitations were also noted, of concern most included studies examined training for roles in mental health, with the balance focusing on consumer rather than carers/family roles. This is likely reflective of both the existing levels of employment for these roles and the lack of training available for these roles. This highlights the critical need for AOD and carer-specific LLE trainings. While the importance of these roles and associated trainings have been emphasized (Chapman et al., 2020), this has not translated into program development or availability. Such training programs are necessary due to the specific challenges encountered in these unique LLE roles.

We also observed adverse outcomes reported by the LLE workforce trainees. Understanding limitations identified by trainees offers opportunities for future improvement of LLE workforce training. Suggestions for future directions are outlined below. Further limitations pertained to conflicting role expectations, negative reactions from others, and limited preparation and support. Our results echo prior findings on the many challenges faced by this workforce and the need to adequately prepare team members who work alongside LLE workers about the LLE role (Davidson et al., 2012; Kemp & Henderson, 2012). Furthermore, our findings are consistent with past observations that a significant challenge for LLE workers in many environments stems from the negative attitudes of clinicians toward this type of work and workplace culture that these attitudes tend to promote (Scanlan et al., 2020).

Meta-analytic evidence has identified adequate LLE training as a central element for the successful employment and integration of individuals with lived experience into the workforce (Walker & Bryant, 2013). One of the most consistent themes in prior literature is that adequate training, for each of the LLE workforces, their multidisciplinary peers, and leaders in their organisations, is critical to the success of LLE workforce initiatives, but it is often lacking. In line with previous suggestions (Chinman et al., 2008; McLean et al., 2009; Scanlan et al., 2020), providing training to the wider organisation, beyond the LLE worker, would likely increase integration and acceptance of LLE workers, while reducing potentially stigmatising non-LEW staff attitudes.

Consistent with existing literature, our findings suggest that training topics, especially those that are aimed at ensuring LLE workers are safe and that boundaries are respected, can enhance the legitimacy of the LLE workforce and facilitate their integration into the mental health system (Kilpatrick et al., 2017). Similar to our findings, research has demonstrated that the most successful training components in engaging peers in service provision roles appear to include the right combination of information (educational content) and skills-based content (e.g., communication) (Mitra & Globerman, 2013).

Moreover, to ensure the sustainability of LLE work, content related to self-care and self-disclosure through meaningful and safely structured sharing of personal stories is key. Many of these content areas are consistent with prior literature noting the importance of their inclusion in initial training programs for peer support workers (Charles et al., 2021). Future trainings that include these components may optimise positive outcomes for trainees. Ideally, adequate coverage of important topics and content areas would be ensured through the development of standardised processes for curriculum development and LLE workforce accreditation (Charles et al., 2021).

A large proportion of the training was delivered in person. Only a few studies examined training provided via online platforms. In light of the COVID-19 climate whereby online delivery of a variety of adult education and training and other types of interventions has become routine, online provision of training has become more acceptable and indeed an expected avenue for improved accessibility. A recent Delphi study examining the feasibility of online delivery of training topics for peer support workers found online delivery to be acceptable with the caveat that blended modalities were used, including some face-to-face learning or concurrent in-person support from a trainer (Charles et al., 2021). Greater availability of self-paced learning online and hybrid trainings may increase access otherwise limited by geographic or other constraints such as time constraints and ongoing life challenges faced by this workforce and provide opportunities for trainees to catch up on missed sessions (Colon et al., 2010; Deren et al., 2012; Treloar et al., 2012). The provision of diverse trainings is needed to accommodate the unique needs of the individual LLE worker. For example, specific training pertaining to organisation readiness and integration, allowing for a more seamless transition to the workplace (Our Future Project Partnership, 2021). Additionally, follow-up support at a personal level appears essential to assist trainees experiencing mental health issues which were commonplace among trainees, or other challenges (Colon et al., 2010; Deren et al., 2012; Hegedüs et al., 2016; Wolf, 2014).

### Priority Foci for Future Training

In light of the positive impacts and limitations of training programs identified in this review, several recommendations emerge for enhancing training outcomes for the LLE workforce. Attention to these combined components is likely to enhance quality and potential of training for these workforces.

1. Trainee wellbeing throughout is the highest priority. Training at all stages of professional development would emphasise and support trainees’ own health and self-care (Chapman et al., 2018).
2. The unique contributions of LLE workers remain central to training and subsequent workforce roles (Meehan et al., 2002). In this vein, training programs should be designed and delivered by or with a team of service user and carer/family workers who optimally have experience as educators. Program co-production would likely result in colleagues of LLE workers having awareness and understanding of the need for shared power and how this can effectively be achieved. This could include ensuring LLE workers receive training to promote confidence and competence to meaningfully contribute to their workplace and engage with those in leadership positions.
3. Preparation for integration into the workplace and ongoing professional development should include practical training such as supervised placements to provide opportunity to safely apply newly acquired skills in the workplace. Once employed, ongoing support during and beyond the formal learning period is key, through individual, peer, and group supervision (Stockmann et al., 2019).
4. Facilitation of a culture of acceptance for the LLE workforce is crucial and can be supported by ensuring roles are well-defined and clearly positioned within multidisciplinary teams and by providing suitable induction to the new workplace and practices required in LLE workforce roles (Byrne et al., 2021b; Franke et al., 2010; Hegedüs et al., 2016; Meehan et al., 2002; Vandewalle et al., 2016; Zeng & McNamara, 2021).
5. Post-induction training content needs as much attention as pre-induction. Ongoing support needs to align with expectations for the LLE roles, giving emphasis to “real world” content, and balance didactic and experiential learning approaches to assist skill translation and consolidation of theoretical material (Atif et al., 2019; Bentley, 2000).
6. Providing training for staff beyond the LLE worker, such as to management, team leaders, and human resource staff (Our Future Project Partnership, 2021). Educating broader organisational members of the key role of the LLE worker would likely result in necessary organisational shifts wherein the LLE worker is valued and understood. This would result in a safe and supporting workplace where LLE training skills and experiential knowledge can be meaningfully applied.
7. Finally, the complexity of role duality needs to be managed well, specifically the shift from or between being a help seeker with personal experiences of mental health and/or AOD issues to being a helper who supports others with those issues (Hegedüs et al., 2016).

### Future Directions for Research

The majority of studies identified in this review examined service user focused trainings for mental health settings. In light of this, further research is required to understand the impact of training for carer/family lived experience workforce roles and for training in the AOD sector. Training was also predominantly for standalone, short-term projects highlighting the need for more research on community-based trainings for ongoing roles. Only two of the identified studies reported on randomized controlled trials. Further research using this methodology is needed to provide higher level evidence for the impact of training. Evidence for impact of training would be further strengthened by research examining clinical outcomes for end-users from LLE interventions in mental health and AOD settings as related to particular training approaches.

### Strengths and Limitations

This is the first study to systematically review peer-reviewed literature examining empirical findings related to trainings for service user and carer/family LLE workforce roles within the context of mental health and AOD settings. In doing so, it sheds light on pertinent content and methods aligned with positive outcomes from LLE workforce training. However, there are several limitations. The diversity of study designs created challenges in identifying direct associations between content and outcomes, although general patterns could be observed. While this review reflects developments in LLE workforce trainings, it was restricted to papers conducted since 2000 onwards. Further, grey literature was excluded from the review and it is possible that literature on this topic remains unpublished.

### Conclusion

Findings from this rapid review illustrate that positive outcomes are typically achieved for professional and personal development from participation in LLE workforce training, although limitations of training were also apparent. Positive outcomes related to the presence of key components in the training and inclusion of these components in future training packages is indicated, together with addressing existing gaps in diversity of methods and content for induction and follow up training. These proposed directions will be strengthened through LLE leadership in development of any future training.

## Supplementary Information

Below is the link to the electronic supplementary material.

Supplementary file1 (DOCX 37 KB)

Supplementary file2 (DOCX 31 KB)

Supplementary file3 (DOCX 38 KB)
