# Supplementary material for: Training of Lived Experience Workforces: A Rapid Review of Content and Outcomes
Source: Adm Policy Ment Health. 2022 Nov 10;50(2):177–211. doi: 10.1007/s10488-022-01232-z (PMC9648875; doi:10.1007/s10488-022-01232-z)
Supplement: Supplementary file 2 — Supplementary file2 (DOCX 31 KB) [file 10488_2022_1232_MOESM2_ESM.docx]

Supplementary 2. Database search terms and search strategy.

## CINAHL

| **Search ID#** | **Search Terms & Strategy** | **Results** |
| --- | --- | --- |
| S1 | Peer counselling (MeSH) | 1076 |
| S2 | Peer assistance programs (MeSH) | 961 |
| S3 | ((peer* OR consumer* OR carer* OR “lived experience”) **NEAR/3** (workforce OR worker* OR “support worker*”)) | 7603 |
| S4 | “expert* by experience” | 381 |
| S5 | “family caregiver*” | 7677 |
| S6 | S1 OR S2 OR S3 OR S4 OR S5 | 17256 |
| S7 | Staff development (MeSH) | 27955 |
| S8 | Training | 236135 |
| S9 | Education | 700984 |
| S10 | Development | 560818 |
| S11 | Course* | 130343 |
| S12 | Online education (MeSH) | 1137 |
| S13 | “e learning” | 8679 |
| S14 | “capacity building” | 2593 |
| S15 | S7 OR S8 OR S9 OR S10 OR S11 OR S12 OR S13 OR S14 | 1345634 |
| S16 | Mental health (MeSH) | 42398 |
| S17 | Mental health services (MeSH) | 35107 |
| S18 | “mental health” | 156557 |
| S19 | Mental disorders (MeSH) | 60617 |
| S20 | Behavioral and mental disorders (MeSH) | 345 |
| S21 | “mental* ill*” | 28908 |
| S22 | “mental disorder*” | 74616 |
| S23 | “behavio*ral health” | 5930 |
| S24 | Alcohol* | 110759 |
| S25 | Alcoholism (MeSH) | 16542 |
| S26 | Alcohol related disorders (MeSH) | 3053 |
| S27 | Alcohol abuse (MeSH) | 11070 |
| S28 | Substance abuse (MeSH) | 31960 |
| S29 | Substance dependence (MeSH) | 9919 |
| S30 | Substance use disorders (MeSH) | 39763 |
| S31 | ((substance OR drug OR alcohol) **NEAR/1** (use OR abuse OR dependence OR addiction OR disorder*)) | 631388 |
| S32 | S16 OR S17 OR 18 OR S19 OR S20 OR S21 OR S22 OR S23 OR S24 OR S25 OR S26 OR S27 OR S28 OR S29 OR S30 OR S31 | 849764 |
| S33 | S6 AND S15 AND S32 | 1218 |
| S34 | S6 AND S15 AND S32 limit to year 2000-2021 | 1142 |
| S35 | S6 AND S15 AND S32 limit to peer reviewed | 1046 |
| S36 | S6 AND S15 AND S32 limit to English language | 1028 |

## *Note*. MeSH = Medical Subject Headings.

## Medline

| **Search ID#** | **Search Terms & Strategy** | **Results** |
| --- | --- | --- |
| S1 | Peer counselling | 281 |
| S2 | Peer assistance programs | 9 |
| S3 | ((peer* OR consumer* OR carer* OR “lived experience”) ADJACENCY/3 (workforce OR worker* OR “support worker*”)) | 1075 |
| S4 | “expert* by experience” | 133 |
| S5 | “family caregiver*” | 8327 |
| S6 | OR/1-5 | 9813 |
| S7 | Staff development (MeSH) | 9651 |
| S8 | Training | 487934 |
| S9 | Education | 955991 |
| S10 | Development | 2906781 |
| S11 | Learning | 440606 |
| S12 | Course* | 643519 |
| S13 | “e learning” | 3262 |
| S14 | “capacity building” | 7126 |
| S15 | OR/7-14 | 4787670 |
| S16 | Mental health (MeSH) | 45039 |
| S17 | Mental health services (MeSH) | 35611 |
| S18 | “mental health” | 216106 |
| S19 | Mental disorders (MeSH) | 168109 |
| S20 | Behavioral and mental disorders | 12908 |
| S21 | “mental* ill*” | 50723 |
| S22 | “mental disorder*” | 208737 |
| S23 | Behavio?ral health | 6922 |
| S24 | Alcohol* | 446068 |
| S25 | Alcoholism (MeSH) | 76692 |
| S26 | Alcohol-related disorders (MeSH) | 5459 |
| S27 | Substance-related disorders (MeSH) | 98655 |
| S28 | ((substance OR dug OR alcohol) ADJACENCY/1 (abuse OR dependence OR addiction OR disorder* OR “use”)) | 195681 |
| S29 | OR/16-28 | 942641 |
| S30 | 6 AND 16 AND 29 | 573 |
| S31 | Limit 30 to (English language and year=2000-current) | 510 |

## *Note*. MeSH = Medical Subject Headings.

## PsycINFO

| **Search ID#** | **Search Terms & Strategy** | **Results** |
| --- | --- | --- |
| S1 | Peer counselling (MeSH) | 1162 |
| S2 | Peer assistance program* | 19 |
| S3 | ((peer* OR consumer* OR carer* OR “lived experience”) ADJACENCY/3 (workforce OR worker* OR “support worker*”)) | 723 |
| S4 | “expert* by experience” | 140 |
| S5 | “family caregiver*” | 6466 |
| S6 | OR/1-5 | 8455 |
| S7 | professional development (MeSH) | 66009 |
| S8 | Training | 298863 |
| S9 | Education | 499531 |
| S10 | Development | 851031 |
| S11 | Learning | 501227 |
| S12 | Course* | 204056 |
| S13 | “e learning” | 3456 |
| S14 | “capacity building” | 2405 |
| S15 | OR/7-14 | 1783450 |
| S16 | Mental health (MeSH) | 73101 |
| S17 | Mental health services (MeSH) | 43399 |
| S18 | “mental health” | 236472 |
| S19 | Mental disorders (MeSH) | 897382 |
| S20 | Behavioral and mental disorders | 14382 |
| S21 | “mental* ill*” | 62059 |
| S22 | “mental disorder*” | 178675 |
| S23 | Behavio?ral health | 7798 |
| S24 | Alcohol* | 150838 |
| S25 | Alcoholism (MeSH) | 31131 |
| S26 | Alcohol abuse (MeSH) | 49665 |
| S27 | Drug abuse (MeSH) | 49141 |
| S28 | Drug dependency (MeSH) | 12955 |
| S29 | Substance use disorder (MeSH) | 134824 |
| S30 | ((substance OR drug OR alcohol) ADJACENCY/1 (“use” OR abuse OR dependence OR addiction OR disorder*)) | 181989 |
| S31 | OR/16-30 | 1192838 |
| S32 | 6 AND 15 AND 31 | 1435 |
| S33 | Limit 32 to (peer reviewed journal and English language and year=”2000-Current”) | 906 |

## *Note*. MeSH = Medical Subject Headings.

##

## Web Of Science

| **Search ID#** | **Search Terms & Strategy** | **Results** |
| --- | --- | --- |
| S1 | **((((TS=("peer counselling" OR "peer assistance program*" )) OR TS=(((peer* OR consumer* OR carer* OR "lived experience") NEAR/3 (workforce OR worker* OR "support worker*")))) OR TS=("expert* by experience" OR "family caregiver*")) AND TS=(training OR education OR development OR course* OR "e learning" OR "capacity building")) AND TS=("mental health" OR "mental disorder*" OR "mental* ill*" OR "mental disorder*" OR "behavio*ral health" OR alcohol* OR ((substance OR drug OR alcohol) NEAR/1 ("use" OR abuse OR dependence OR addiction OR disorder*)))** | 1378 |
| S2 | Refined by languages: English | 1293 |
| S3 | Refined by publication years 2000-2021 | 1228 |
