# Supplementary material for: Training of Lived Experience Workforces: A Rapid Review of Content and Outcomes
Source: Adm Policy Ment Health. 2022 Nov 10;50(2):177–211. doi: 10.1007/s10488-022-01232-z (PMC9648875; doi:10.1007/s10488-022-01232-z)
Supplement: Supplementary file 3 — Supplementary file3 (DOCX 38 KB) [file 10488_2022_1232_MOESM3_ESM.docx]

Supplementary 3. Quality Assessment of Included Studies (*N* = 36).

| **Study** | **1** | **2** | **3** | **4** | **5** | **6** | **7** | **8** | **9** | **10** | **11** | **12** | **13** | **14**† | **Total with 14** | **Total with 14 excluded** |
| --- | --- | --- | --- | --- | --- | --- | --- | --- | --- | --- | --- | --- | --- | --- | --- | --- |
| Atif (2019)* - Pakistan | 3 | 3 | 3 | 3 | 3 | 3 | 3 | 3 | 3 | 3 | 3 | 3 | 3 | 2 | 41 | 39 |
| Bentley 2000 - USA | 3 | 3 | 2 | 3 | 2 | 2 | 3 | 3 | 3 | 3 | 3 | 2 | 1 | 0 | 33 | 33 |
| Blixen 2015^ - USA | 3 | 3 | 3 | 3 | 3 | 3 | 3 | 3 | 3 | 3 | 3 | 3 | 3 | 2 | 41 | 39 |
| Cleary 2009 - Australia | 3 | 3 | 3 | 3 | 3 | 3 | 3 | 3 | 2 | 3 | 3 | 2 | 3 | 0 | 37 | 37 |
| Colon (2010)^ - USA | 3 | 3 | 3 | 3 | 3 | 3 | 3 | 3 | 2 | 3 | 3 | 3 | 0 | 2 | 37 | 35 |
| Compton (2013) - USA | 3 | 3 | 2 | 3 | 2 | 3 | 3 | 3 | 3 | 2 | 3 | 3 | 3 | 0 | 36 | 36 |
| Crisanti 2016 - USA | 3 | 3 | 1 | 3 | 2 | 2 | 3 | 3 | 3 | 3 | 3 | 3 | 3 | 0 | 35 | 35 |
| Cronise 2016 - USA | 3 | 3 | 3 | 3 | 3 | 3 | 3 | 3 | 3 | 3 | 3 | 3 | 3 | 0 | 39 | 39 |
| Cunningham 2020 - USA | 3 | 3 | 3 | 3 | 3 | 2 | 1 | 3 | 3 | 3 | 3 | 1 | 3 | 0 | 34 | 34 |
| Deren 2011* -USA | 3 | 3 | 3 | 3 | 3 | 3 | 3 | 3 | 3 | 3 | 3 | 3 | 3 | 2 | 41 | 39 |
| Franke (2010)* - Australia | 3 | 3 | 3 | 3 | 3 | 2 | 3 | 2 | 3 | 1 | 3 | 2 | 0 | 0 | 31 | 31 |
| Gammonley 2001 – Unknown | 3 | 3 | 1 | 3 | 3 | 3 | 3 | 3 | 3 | 3 | 3 | 1 | 2 | 0 | 34 | 34 |
| Gerry 2011^ - UK (England) | 2 | 2 | 2 | 3 | 2 | 2 | 3 | 3 | 2 | 3 | 3 | 2 | 2 | 0 | 31 | 31 |
| Hegedüs (2016)* - Switzerland | 3 | 3 | 2 | 3 | 3 | 3 | 3 | 3 | 3 | 3 | 3 | 3 | 3 | 0 | 38 | 38 |
| Hegedüs (2021) - Switzerland and Germany | 3 | 3 | 2 | 3 | 3 | 3 | 3 | 3 | 3 | 3 | 3 | 3 | 3 | 0 | 38 | 38 |
| Hoagwood (2018) - USA | 3 | 3 | 3 | 3 | 3 | 3 | 3 | 3 | 3 | 3 | 3 | 3 | 3 | 0 | 39 | 39 |
| Horwitz (2020) - USA | 3 | 3 | 2 | 3 | 2 | 3 | 3 | 3 | 3 | 3 | 3 | 2 | 3 | 0 | 36 | 36 |
| Joo (2018) - USA | 3 | 3 | 3 | 3 | 3 | 3 | 3 | 3 | 3 | 3 | 3 | 3 | 3 | 0 | 39 | 39 |
| King (2009)^ - Australia | 3 | 3 | 3 | 3 | 3 | 3 | 3 | 3 | 3 | 3 | 3 | 3 | 3 | 0 | 39 | 39 |
| Meehan (2002)* Australia | 3 | 3 | 3 | 3 | 3 | 3 | 3 | 3 | 3 | 3 | 3 | 2 | 1 | 0 | 36 | 36 |
| Olin (2010) - USA | 3 | 3 | 3 | 3 | 3 | 3 | 3 | 3 | 3 | 3 | 3 | 3 | 3 | 0 | 39 | 39 |
| Rapp (2008) - USA | 2 | 2 | 3 | 3 | 3 | 3 | 3 | 3 | 3 | 3 | 3 | 3 | 3 | 0 | 37 | 37 |
| Rodriguez (2011) - USA | 3 | 3 | 3 | 3 | 3 | 3 | 3 | 3 | 3 | 2 | 3 | 3 | 3 | 0 | 38 | 38 |
| Sanchez-Moscona 2021^ - Spain | 3 | 3 | 3 | 3 | 2 | 3 | 3 | 3 | 3 | 3 | 3 | 3 | 1 | 0 | 36 | 36 |
| Simpson (2014) * - UK (England) | 3 | 3 | 3 | 3 | 3 | 3 | 3 | 3 | 3 | 3 | 3 | 3 | 1 | 0 | 37 | 37 |
| Stewart 2008^ - Australia | 3 | 3 | 3 | 3 | 3 | 2 | 2 | 3 | 3 | 2 | 3 | 2 | 1 | 0 | 33 | 33 |
| Stockmann 2019^ - UK | 3 | 3 | 2 | 3 | 3 | 3 | 3 | 3 | 3 | 3 | 3 | 3 | 3 | 0 | 38 | 38 |
| Stoneking 2007 - USA | 3 | 3 | 3 | 3 | 3 | 3 | 3 | 3 | 3 | 3 | 3 | 3 | 2 | 0 | 38 | 38 |
| Toikko 2016^ - Finland | 3 | 3 | 3 | 3 | 2 | 3 | 3 | 3 | 3 | 3 | 3 | 0 | 2 | 0 | 34 | 34 |
| Treloar 2012^ - Australia | 3 | 3 | 3 | 3 | 3 | 3 | 3 | 3 | 3 | 3 | 3 | 3 | 3 | 0 | 39 | 39 |
| Tsai 2017 - USA | 3 | 3 | 3 | 3 | 3 | 3 | 3 | 3 | 3 | 3 | 3 | 3 | 3 | 0 | 39 | 39 |
| Tse (2014)* - Hong Kong | 3 | 3 | 2 | 3 | 2 | 3 | 3 | 3 | 3 | 3 | 3 | 3 | 3 | 0 | 37 | 37 |
| Weeks (2006)* - USA | 3 | 3 | 3 | 3 | 3 | 3 | 3 | 3 | 3 | 3 | 3 | 3 | 2 | 0 | 38 | 38 |
| Willging (2016)^ - USA | 3 | 3 | 3 | 3 | 3 | 3 | 3 | 3 | 3 | 3 | 3 | 3 | 3 | 2 | 41 | 39 |
| Wolf 2014 - USA | 3 | 3 | 3 | 2 | 3 | 2 | 2 | 3 | 3 | 2 | 1 | 3 | 1 | 0 | 31 | 31 |
| Wolfe 2013 - USA | 3 | 3 | 3 | 3 | 3 | 3 | 3 | 3 | 3 | 3 | 3 | 3 | 3 | 0 | 39 | 39 |
| **AVERAGE** | 2.94 | 2.94 | 2.67 | 2.97 | 2.78 | 2.81 | 2.89 | 2.97 | 2.92 | 2.83 | 2.94 | 2.61 | 2.36 | 0.22 | **36.92** | **36.64** |

*Note*. Items 1-13: 0 = not reported; 1 = reported but inadequate; 2 = reported and partially adequate; 3 = sufficiently reported. Description of Quality Assessment for Diverse Studies (QuADS) quality assessment criteria: 1) Theoretical or conceptual underpinnings to the research; 2) Statement of research aim/s; 3) Clear description of research setting and target population; 4) The study design is appropriate to address the stated research aim/s; 5) Appropriate sample to address the research aim/s; 6) Rationale for choice of data collection tool/s; 7) The format and content of data collection tool is appropriate to address the stated research aim/s; 8) Description of data collection procedure; 9) Recruitment data provided; 10) Justification for analytic method selected; 11) The method of analysis was appropriate to answer the research aim/s; 12) Evidence that the research stakeholders have been considered in research design or conduct; 13) Strengths and limitations critically discussed; 14) Study described as randomised and method of randomisation appropriate. † = item from Jadad scale (item 14): Was the study described as randomized? 0 = not reported; 1 = described as randomised but method not described or inappropriate; 2 = described as randomised with appropriate method of randomisation used. * = mixed-methods studies; ^ = qualitative studies; all remaining studies are quantitative in design.
